# Influence of BMI on Gait Characteristics of Young Adults: 3D Evaluation Using Inertial Sensors

**DOI:** 10.3390/s19194221

**Published:** 2019-09-28

**Authors:** Valeria Rosso, Valentina Agostini, Ryo Takeda, Shigeru Tadano, Laura Gastaldi

**Affiliations:** 1 Department of Mechanical and Aerospace Engineering, Politecnico di Torino, Italy, Corso Duca degli Abruzzi 24, 10129 Torino, Italy; 2 Department of Electronics and Telecommunications, Politecnico di Torino, Corso Duca degli Abruzzi 24, 10129 Torino, Italy; valentina.agostini@polito.it; 3 Division of Human Mechanical Systems and Design, Faculty of Engineering, Hokkaido University, Sapporo, Hokkaido 060-8628, Japan; r.takeda@eng.hokudai.ac.jp (R.T.); tadano@eng.hokudai.ac.jp (S.T.); 4 National Institute of Technology, Hakodate College, Hakodate 042-8501, Japan; 5 Department of Mathematical Sciences, Politecnico di Torino, Corso Duca degli Abruzzi 24, 10129 Torino, Italy

**Keywords:** wearable sensors, inertial sensors, gait analysis, spatio-temporal parameters, joint kinematics, young adults, overweight, obese

## Abstract

Overweight/obesity is a physical condition that affects daily activities, including walking. The main purpose of this study was to identify if there is a relationship between body mass index (BMI) and gait characteristics in young adults. 12 normal weight (NW) and 10 overweight/obese (OW) individuals walked at a self-selected speed along a 14 m indoor path. H-Gait system, combining seven inertial sensors (fixed on pelvis and lower limbs), was used to record gait data. Walking speed, spatio-temporal parameters and joint kinematics in 3D were analyzed. Differences between NW and OW and correlations between BMI and gait parameters were evaluated. Conventional spatio-temporal parameters did not show statistical differences between the two groups or correlations with the BMI. However, significant results were pointed out for the joint kinematics. OW showed greater hip joint angles in frontal and transverse planes, with respect to NW. In the transverse plane, OW showed a greater knee opening angle and a shorter length of knee and ankle trajectories. Correlations were found between BMI and kinematic parameters in the frontal and transverse planes. Despite some phenomena such as soft tissue artifact and kinematics cross-talk, which have to be more deeply assessed, current results show a relationship between BMI and gait characteristics in young adults that should be looked at in osteoarthritis prevention.

## 1. Introduction

The World Health Organization defined the grade of overweight and obesity in adults using the Body Mass Index (BMI) as follows: normal weight (18.5 < BMI < 24.5), overweight (25.0 < BMI < 29.9), and obese (BMI > 30.0) [1]. It is well known that the overweight and/or obesity condition has functional implication on everyday life. In particular, it has been demonstrated that extra weight alters the normal mechanism of gait, reducing the hip and knee flexion in stance phase [2,3], increasing the ankle plantarflexion in stance, and the ankle dorsiflexion in swing [2]. In obese adults, walking speed is reduced and gait is characterized by shorter step length, lower step frequency, and longer stance phase [4]. In addition, obesity is generally correlated with knee and hip osteoarthritis [5] due to the different joint load distribution. The effects of overweight and obesity on gait kinematics in adults are well known. However, there is a lack of knowledge on gait data from young overweight/obese adults. Nevertheless, the early detection of gait deviations from normality in a population of overweight/obese young adults might be very important in osteoarthritis prevention [6]. For example, it could suggest preventive therapies to this population.

Gait analysis is extensively adopted to evaluate human locomotion [7,8,9] and to monitor its changes due to ageing [10], disease [11,12,13], or rehabilitation [14,15]. Optoelectronic systems are a gold standard in biomechanical assessment of human gait [16]. However, these systems have few drawbacks. Optoelectronic systems are expensive, data collection is usually limited to a small volume, and data analysis requires a long amount of time. For these reasons, wearable sensors composed of accelerometers and gyroscopes are increasingly adopted to evaluate human motion in everyday life, sport, and clinical purposes when the use of stereophotogrammetric systems is challenging [17]. Wearable sensors can be placed in different anatomical segments and arranged in different configurations [18,19,20].

Wearable inertial sensors have already been used in human gait analysis, showing good results in application on both healthy adults [19] and healthy elderly [21], but also on pathological population such as Parkinson’s disease [22] and cerebral palsy [18]. The majority of the studies focused on the evaluation of human gait in terms of walking speed and spatio-temporal parameters [19,23,24]. Besides spatio-temporal parameters, lower limb joint kinematics also provides crucial information about human gait characteristics. Pelvis movements and hip, knee, and ankle joint kinematics are largely evaluated using inertial sensors [25]. The majority of the work in this field focused on flexion-extension movements in the sagittal plane. However, the analysis in the frontal plane (abduction/adduction) and in the transverse plane (internal/external rotation) can also provide relevant information. Tadano et al. [6] showed that the knee and ankle kinematics of osteoarthritic patients is different in the transverse plane compared to control group of healthy subjects. Despite this evidence, a limited number of studies assessed 3D kinematics of lower limbs using wearable inertial sensors.

Therefore, the purpose of the current work was to compare gait characteristics of overweight/obese (OW) and normal weight (NW) subjects. In particular, the current study aims to answer the question of if there a relationship between BMI and gait biomechanics in young adults. This study was conducted analyzing spatio-temporal parameters and joint kinematics in the three planes. In addition, other kinematic parameters of knee and ankle joints were proposed and evaluated. We hypothesize that NW and OW young subjects show different values for spatio-temporal and kinematic data.

## 2. Materials and Methods

### 2.1. Subjects

A total of 22 healthy young males (age: 26 ± 1.8 years, height: 178 ± 8.5 cm) were recruited for the current study. Subjects were divided into two groups according to their BMI [1]. 12 subjects were included in the NW group (18.5 < BMI < 24.9 kg/m^2^) and 10 were included in the OW group (BMI > 25.0 kg/m^2^). Gait data were taken from retrospective studies performed at Politecnico di Torino, Italy. After being informed about the test procedures and aims, all the subjects signed a consent form. The procedures were performed in accordance with the Declaration of Helsinki. 

### 2.2. Acquisition System

The H-Gait system was used to collect gait data [26]. The H-Gait system includes seven inertial sensors (TSDN121, ATR Promotions). Each sensor is composed of a tri-axial accelerometer (full scale 16 g), a tri-axial gyroscope (full scale 2000 dps), and a tri-axial magnetometer (full scale 1200 μT) [27]. For the current test, accelerometers and gyroscope ranges were set to ±4 g (accuracy: 0.12 mg) and ±500 dps (accuracy: 0.015 dps), respectively. The sampling frequency was set equal to 100 Hz. Acceleration, angular velocity, and magnetic data were collected simultaneously during gait trials and saved locally. Then, they were transferred to a laptop. Magnetometer data were not used for the analysis.

### 2.3. Test Protocol and System Calibration

**Figure 1:**
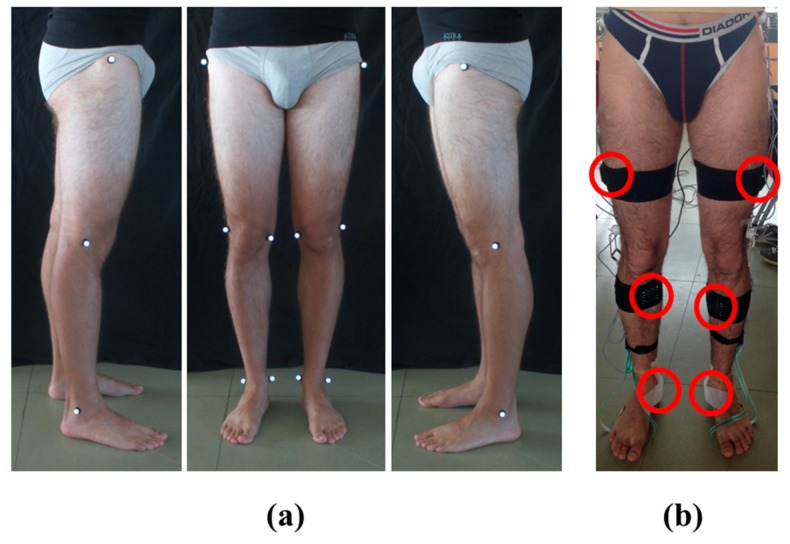
Subject preparation for the protocol: (**a**) Photos from the left, front, and right side of the subject with the reflective markers placed on both lower limbs; (**b**) Placement and fixing of the six sensors on the subject’s lower limbs.

Before starting the gait test, the inertial sensors were positioned on the subject and a calibration procedure was performed to convert data from the sensors coordinate system to the anatomical coordinate systems. The subject preparation and calibration procedure were explained in detail in [26,28]. Briefly, the following steps were carried out in a laboratory:

(1). Anatomical measurements of pelvis breadth, thigh height, shank height, and sphyrion height.
(2). Placement of reflective markers, bilaterally, on the anatomical representative points: greater trochanter, lateral epicondyle of the femur, medial epicondyle of the femur, lateral malleolus and medial malleolus. Three photos were shot from the front, left, and right sides of the subject (Figure 1a). The markers were removed.
(3). Fixing of inertial sensors on subject’s pelvis and both lower limbs using elastic Velcro^®^ bands and medical tape. The sensor on the pelvis was located posteriorly in the middle point between iliac crests. The six sensors on the lower limbs were positioned on the lateral side of the thighs, on the anterior side of the tibia, and below the medial malleolus, bilaterally [6,28,29] (Figure 1b).
(4). Acquisition of H-Gait signals for three seconds, with the subject in sitting posture, and for another three seconds in upright standing posture.

After the calibration procedure the test started in the same laboratory. The test was performed barefoot walking at a self-selected speed on a straight path of 14 m. To become confident with the equipment worn, subjects walked on the straight path forward and backward once. Then, the subjects performed six walking trials (three forward and three backward), but for the current analysis, only the first walked in the forward direction was considered.

### 2.4. Data Analysis

Walking speed and the following spatio-temporal parameters were calculated: step length (cm), step width (cm), stride length (cm), cycle time (s), stance time (% gait cycle), and cadence (stride/min).

Then, the hip, knee, ankle joint kinematics in the sagittal (flexion/extension), frontal (abduction/adduction), and transverse (internal/external rotation) planes were calculated [26,30]. Heel contacts (HC) were calculated by identifying the peaks of the shank angular velocity. Toe off (TO) events were identified considering toe trajectories [28]. Using HC and TO, joint kinematic signals were normalized with respect to the gait cycle. The joint range of motion (ROM) was calculated as the difference between the maximum and minimum angles during each gait cycle.

**Figure 2:**
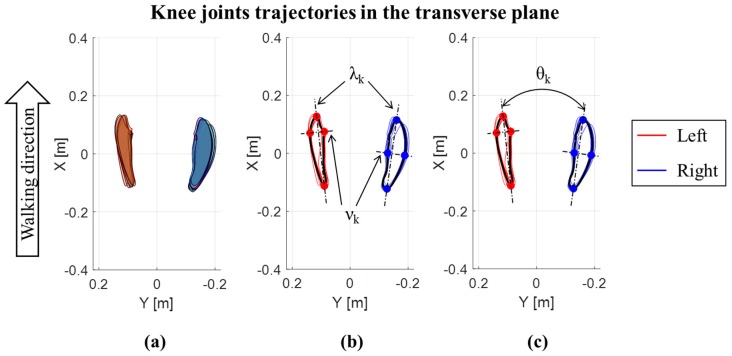
Knee joints trajectories in the transverse plane for an OW subject: (**a**) Area of each left and right step; (**b**) major (λ_k_) and minor (ν_k_) diameters of the average knee trajectories; (**c**) knee opening angle (θ_k_) between the left and right major axes of average knee joint trajectories.

**Figure 3:**
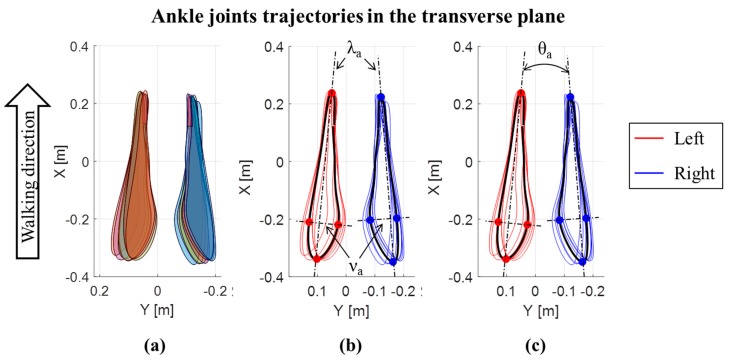
Ankle joints trajectories in the transverse plane for an OW subject: (**a**) Area of each left and right step; (**b**) major (λ_k_) and minor (ν_k_) diameters of the average ankle joints trajectories; (**c**) ankle opening angle (θ_a_) between the left and right major axes of average ankle joint trajectories.

In addition to the joint ROMs in the three planes, the trajectories of the center of the knee and ankle joints in the transverse plane were also evaluated, for each cycle and both the left and right lower limbs [6,31]. Trajectories are expressed with respect to a reference frame fixed on the pelvis, with the X axis directed as the walking direction, the Y axis in the medio-lateral direction and the origin (X = 0; Y = 0) in the middle point between the iliac crests. A representative example of trajectories for an OW subject is reported in Figure 2 for the knee, and for the ankle in Figure 3. For each lower limb and each gait cycle, the area of knee trajectory (A_k_) was calculated (Figure 2a) and averaged across cycles. Then, the area values were averaged between the left and right lower limbs. Furthermore, the average knee trajectories in transverse plane were calculated for each lower limb. The major (λ_k_) and minor (ν_k_) diameters of the average knee trajectories were obtained as shown in Figure 2b. Finally, the knee opening angle (θ_k_) between the left and right major axes were assessed [6,31] (Figure 2c). The same data were calculated for the ankle joint trajectories (Figure 3), being in this case: A_a_ the ankle area, λ_a_ the ankle major diameter, ν_a_ the ankle minor diameter, and θ_a_ the ankle opening angle between the left and right major axes. The trajectory for the hip joint in the transversal plane was also calculated, but it was not represented due to its negligible path in this plane.

The analysis to calculate walking speed, spatio-temporal parameters, and the joint kinematics was performed using a custom made Matlab^®^ script (MatLab^®^ and Release 2018, The MathWorks, Inc., Natick, Massachusetts, United States) For both the spatio-temporal and kinematic analysis, parameters from right and left side were averaged for the statistical analysis.

### 2.5. Statistical Analysis

Non-parametric statistics were used. Statistical differences between NW and OW of anthropometric data (age, height, weight, and BMI) were evaluated using the Mann Whitney test. Statistical differences in joint kinematics, spatio-temporal parameters, and walking speed between NW and OW subjects were also assessed using the Mann Whitney test. Correlations of joint kinematics, spatio-temporal parameters, and walking speed with BMI were evaluated using Spearman correlation. Significance level was set at α = 0.05. Statistical analyses were performed with Matlab^®^ Software.

## 3. Results

Comparing age and height between NW and OW, no statistical differences were found, whereas weight and BMI were higher for OW subjects compared to NW (Table 1).

### 3.1. Spatio-Temporal Parameters

Walking speed and spatio-temporal parameters did not show statistical differences between NW and OW subjects (Table 2). In addition, no significant correlations of the walking speed and spatio-temporal parameters with BMI were found.

### 3.2. Joint Kinematics

Table 3 reports kinematic results and statistical differences when present. Hip abduction/adduction ROM and hip internal/external rotation ROM were significantly greater for OW compared to NW (p < 0.05). Concerning knee joint, θ_k_ was significantly greater for OW compared to NW (p < 0.05). Finally, λ_k_ and λ_a_ were statistically shorter for OW than for NW (p < 0.01).

**Figure 4:**
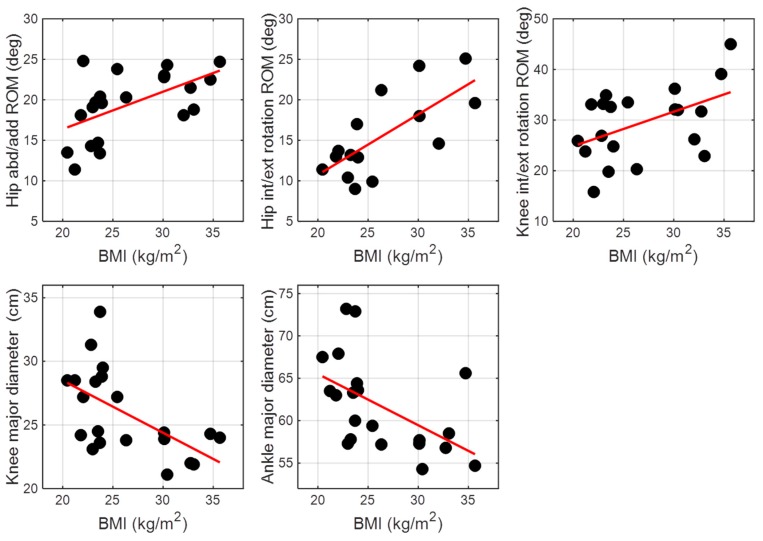
Significant correlations between BMI and joint kinematics parameters.

Concerning the correlations (Figure 4), BMI was positively correlated both with hip abduction/adduction ROM (ρ = 0.56, p < 0.01) and hip internal/external rotation ROM (ρ = 0.71, p < 0.01). For knee joint, BMI was positively correlated with knee internal/external rotation ROM (ρ = 0.51, p < 0.05) and negatively correlated with λ_k_ (ρ = −0.58, p < 0.01). Ankle joint kinematics did not show correlations with BMI in any of the three planes. A negative correlation was found between BMI and λ_a_ (ρ = −0.53, p < 0.05).

## 4. Discussion

Comparing gait characteristics of OW and NW young subjects collected by wearable inertial sensors, this work aimed to assess if there is a correlation between BMI and gait characteristics in young adults, a relationship that can be useful in osteoarthritis prevention. Despite no differences were found in conventional spatio-temporal parameters between OW and NW groups, significant results were pointed out analyzing the joint kinematics. Indeed, OW group showed greater hip abduction/adduction ROM, greater hip internal/external rotation ROM, greater knee opening angle, and shorter major diameters with respect to NW. In addition, correlations were found between the joint kinematics and the BMI. Positive correlations were found between BMI and abduction/adduction ROM of the hip and between BMI and internal/external rotation ROM of the hip and the knee joints. A negative correlation was found between the BMI and the ankle major diameter.

### 4.1. Spatio-Temporal Parameters

Although in the literature there is large consensus about the slower preferred walking speed of obese adults compared to lean adults [32,33,34,35], this is not always the case [36]. Indeed, besides the knee joint load, the energy cost per distance is also an aspect that should be considered when the preferred walking speed is evaluated [36,37]. The energy cost per distance vs. walking speed is a U-shaped curve, with a relatively flat part around 1.2−1.4 m/s [36]. In our study, the self-selected walking speed for both OW and NW subjects is slightly lower compared to the literature results [35], but still close to the range of values with the lowest energy cost per distance expenditure [36,37]. The fact that the walking speed was slightly lower than what it is reported in the literature could be due to the fact that the current data were taken from a retrospective study [28] in which subjects were wearing also a set of electrogoniometers, and this may affect the natural walking speed. Nevertheless, having similar walking speeds between the two groups assured that the changes existing between OW and NW were directly related to the BMI and are not influenced by the walking speed [38].

Concerning the spatial parameters, the literature suggests that OW adults walk with shorter stride length, shorter step length, and larger step width compared to NW adults when walking at a self-selected speed [4]. However, this finding may be directly due to the effect of speed [4]. On the other hand, when OW and NW adults are forced to use the same pre-defined walking speed, no differences were found in stride length [2] and step length [33]. However, it is suggested that OW individuals may alter their gait characteristics when forced to a walking speed that is different from the preferred one [33]. In our study, we found no differences between spatial parameters of OW and NW, although subjects were walking at their self-selected speed. Hence, no gait alterations are expected due to effect of an imposed speed. In this perspective, our tests were conducted in a more ecological condition.

Temporal parameters did not show differences in cycle time, stance time, and cadence. Our results on cadence were in agreement with the literature that reports no differences between OW and NW adults when walking at their preferred speed [35]. The literature showed longer stance time for OW compared to NW adults when walking both at the preferred walking speed [35] and nearly identical speed [2,33]. We found no differences for the stance time. This is consistent with our results of an equal walking speed and step length between groups.

In literature, it was previously suggested that in order to increase their dynamic balance, obese adults tend to reduce walking speed, step length, cadence, and swing phase duration, and to increase step width, stance phase, and double support [32,34]. Considering altogether the results on spatio-temporal parameters (step length and width, cycle and stance time) and walking speed, this suggests that young OW subjects in our study do not show a modified dynamic balance. The subjects enrolled in our study were, on the average, younger and showed a lower BMI with respect to obese adults considered in the other studies [32,34]. Therefore, a combination of young age (which entails gait patterns not affected by degenerative changes) [39] and a relatively lower BMI can explain our spatio-temporal findings in comparison with the existing literature and suggests that conventional spatio-temporal parameters might not reflect potential critical situation that could lead to the development of osteoarthritis.

### 4.2. Joint Kinematics

In the existing literature about lower limb kinematics of overweight/obese adults, most of the studies focused on the sagittal plane. In particular, it was demonstrated that overweight/obese adults have a more erect posture during the stance phase (greater hip extension and ankle plantarflexion, and smaller knee flexion) in order to decrease the knee joint load [33]. We found no difference in joint flexion/extension ROMs between OW and NW subjects. This finding is in accordance with previous studies [2,25,35,40] in which OW and NW subjects did not show any difference in flexion/extension ROM when walking at a velocity close to the self-selected speed obtained in our study. In our previous study [28], a difference between OW and NW was found in hip flexion/extension ROM when the joint kinematics was evaluated using electrogoniometers. This discrepancy was hypothesized to be due to kinematic crosstalk induced by the inertial sensors attached to the pelvis, which was not quantified. The lack of differences in joint flexion/extension ROMs between OW and NW may be related to the BMI of the tested subjects. Indeed, it was previously suggested that it may exists a threshold value of BMI (40 kg/m^2^) above which individuals change their gait to reduce the knee joint load [2,38].

The most important contribution of this study was the assessment of overweight/obese joint kinematics in the frontal and transverse planes, which are usually less considered in gait analysis performed with inertial sensors. Especially in the frontal plane, alterations of the joint kinematics may be one of the causes of lower limb joint injury [41] and osteoarthritis [42]. We found a higher hip abduction/adduction ROM for OW subjects compared to NW, which is in agreement with the existing literature [4,34,35]. We also found a positive correlation between BMI and hip abduction/adduction ROM. These differences between OW and NW subjects could be explained by a higher hip load, a larger thigh girth [34], and a different adductor muscle force during the latter part of the stance phase to better control body sway and upright stability [35,43].

Considering the transverse plane, results showed a positive correlation between BMI and both hip and knee internal/external rotation ROM as well as a larger hip internal/external rotation ROM of OW subjects. We also found a greater knee opening angle for OW compared to NW subjects. Hence, OW subjects tend to widen their knees while advancing. Overall, these findings may be explained not only by a higher hip load and a larger thigh girth of OW subjects [34], but also by a hampered thigh movement in the sagittal plane that has to be compensated in transverse plane [44]. In a previous study, normal weight individuals, both with and without osteoarthritis, were analyzed [6]. For what concerns the knee opening angle, our results are consistent with those of Tadano et al. [6] when considering healthy NW individuals. Despite the close relationship between obesity and osteoarthritis [5], the present work showed a positive knee opening angle in OW subjects, whereas osteoarthritic patients showed a negative opening angle (closing their knee trajectories along the walking direction) [6]. This difference may be explained by the fact that OW subjects from our study did not report any knee pain, thus allowing us to exclude knee osteoarthritis. The OW subjects were also younger compared to the osteoarthritic patients of that study [6]. In addition, differently from our OW population, the osteoarthritic patients of Tadano et al. [6] had a normal BMI. Hence, the populations analyzed in these two studies cannot be directly compared.

We found that both knee and ankle major diameters were shorter in OW compared to NW subjects. This is consistent with slightly shorter step length for OW with respect to NW subjects, even though this difference is not statistically significant. Accordingly, we also found a negative correlation between BMI and both knee and ankle major diameters. The differences between OW and NW pointed out by the 3D joint kinematics suggest that the proposed parameters might sufficiently highlight potential critical situation.

### 4.3. Limitation

Among the recruited individuals, the highest BMI was 34.7 kg/m^2^, which belonged to the obesity class I (as defined by the World Health Organization) [1], but no individuals representative of the obesity class II and class III were included. Although significant differences already emerged from our study, the inclusion of other obesity classes would probably complement current findings on the correlation between BMI and gait biomechanics in young adults. In addition, in order to generalize current results, additional analysis including both genders should be conducted.

Data were from a retrospective study in which subjects were wearing inertial sensors and electrogoniometers as well. Wearing both collecting systems may influence the subjects’ walking speed, slightly reducing it as results showed. This can be identified as a second limitation for this study.

Soft tissue artifact and kinematic cross-talk are still debating topics in the gait analysis literature, especially for obese subjects. In order to reduce the soft tissue artifacts, the sensors were fixed over the bones instead of the muscles [16,45] when possible in this study. However, not investigating soft tissue artifact and kinematic cross-talk could identify a third limitation of this study. In addition, data collection in transverse plane has been validated for normal weight subjects [26], but not for overweight/obese subjects. Therefore, further analysis should be conducted in that sense.

## 5. Conclusions

In order to answer the question of if there is a relationship between BMI and gait biomechanics in young adults, wearable inertial sensors were used to collect gait of overweight/obese and normal weight individuals in this study. Similar walking speeds and spatio-temporal results were found between the two groups. In contrast, joint kinematic parameters allowed identifying a relationship between BMI and gait characteristics in young adults. Moreover, inertial sensors proved useful in assessing subtle differences in all the trajectory planes. This study highlights the importance of also assessing the frontal and transverse planes to fully evaluate the human gait, especially in the early stages of potential critical situations such as overweight or obesity that may lead to the later development of osteoarthritis. 

## Figures and Tables

**Table 1:** Anthropometric data (mean ± standard deviation).

Parameter	NW	OW	*p* Value
Age (years)	26 ± 1.5	26 ± 2.2	0.92
Height (cm)	180.0 ± 8.8	175.3 ± 7.6	0.16
Weight (kg)	74.3 ± 8.2	95.5 ± 12.4	<0.001*
BMI (kg/m^2^)	22.7 ± 1.2	31.1 ± 3.3	<0.001*

Note: * Statistically significant differences between NW and OW.

**Table 2:** Spatio-temporal parameters (mean ± standard deviation).

Parameter	NW	OW	*p* Value
Walking speed (m/s)	1.1 ± 0.1	1.0 ± 0.2	0.50
Step length (cm)	62.4 ± 5.1	56.5 ± 4.2	0.07
Step width (cm)	18.3 ± 9.0	18.7 ± 4.1	0.94
Stride length (cm)	123.5 ± 10.0	113.0 ± 8.3	0.10
Cycle time (s)	1.2 ± 0.2	1.1 ± 0.1	0.11
Stance time (% gait cycle)	55 ± 2	57 ± 2	0.14
Cadence (stride/min)	51.6 ± 4.9	53.8 ± 2.9	0.16

**Table 3:** Joint kinematics parameters (mean ± standard deviation).

Parameter		NW	OW	p Value
Hip	Flexion/extension ROM (deg)	34.7 ± 3.3	32.7 ± 3.4	0.16
	Abduction/adduction ROM (deg)	17.2 ± 4.0	22.0 ± 2.3	0.01*
	Internal/external rotation ROM (deg)	12.6 ± 2.4	18.9 ± 5.4	0.02*
Knee	Flexion/extension ROM (deg)	60.2 ± 6.7	60.7 ± 8.1	0.87
	Abduction/adduction ROM (deg)	15.2 ± 3.9	15.3 ± 4.4	0.86
	Internal/external rotation ROM (deg)	27.1 ± 6.4	32.3 ± 9.9	0.24
	Trajectory area, A_k_ (cm^2^)	142.2 ± 44.4	133.8 ± 45.1	0.59
	Major diameter, λ_k_ (cm)	26.6 ± 3.6	23.9 ± 2.8	0.01*
	Minor diameter, ν_k_ (cm)	6.8 ± 1.9	7.8 ± 2.2	0.43
	Opening angle, θ_k_ (deg)	5.3 ± 13.2	20.2 ± 9.8	0.02*
Ankle	Flexion/extension ROM (deg)	17.9 ± 3.6	18.1 ± 5.2	0.64
	Abduction/adduction ROM (deg)	12.6 ± 2.6	15.2 ± 3.4	0.12
	Internal/external rotation ROM (deg)	19.4 ± 5.4	19.4 ± 2.0	0.76
	Trajectory area, A_a_ (cm^2^)	402.2 ± 112.3	361.7 ± 80.3	0.34
	Major diameter, λ_a_ (cm)	63.8 ± 5.2	57.0 ± 2.2	0.01*
	Minor diameter, ν_a_ (cm)	9.2 ± 2.7	10.1 ± 1.4	0.32
	Opening angle, θ_a_ (deg)	−10.1 ± 8.7	−5.4 ± 12.9	0.56

Note: * Statistically significant differences between NW and OW.

## Abbreviations

The following abbreviations are used in the manuscript:

A_a_	Area of ankle trajectory
A_k_	Area of knee trajectory
BMI	Body Mass Index
HC	Heel contact
NW	Normal weight
OW	Overweight/obese
ROM	Range of motion
TO	Toe off
θ_a_	Ankle opening angle between the left and right major axes
θ_k_	Knee opening angle between the left and right major axes
λ_a_	Major diameters of the average ankle trajectories
λ_k_	Major diameters of the average knee trajectories
ν_a_	Minor diameters of the average ankle trajectories
ν_k_	Minor diameters of the average knee trajectories
